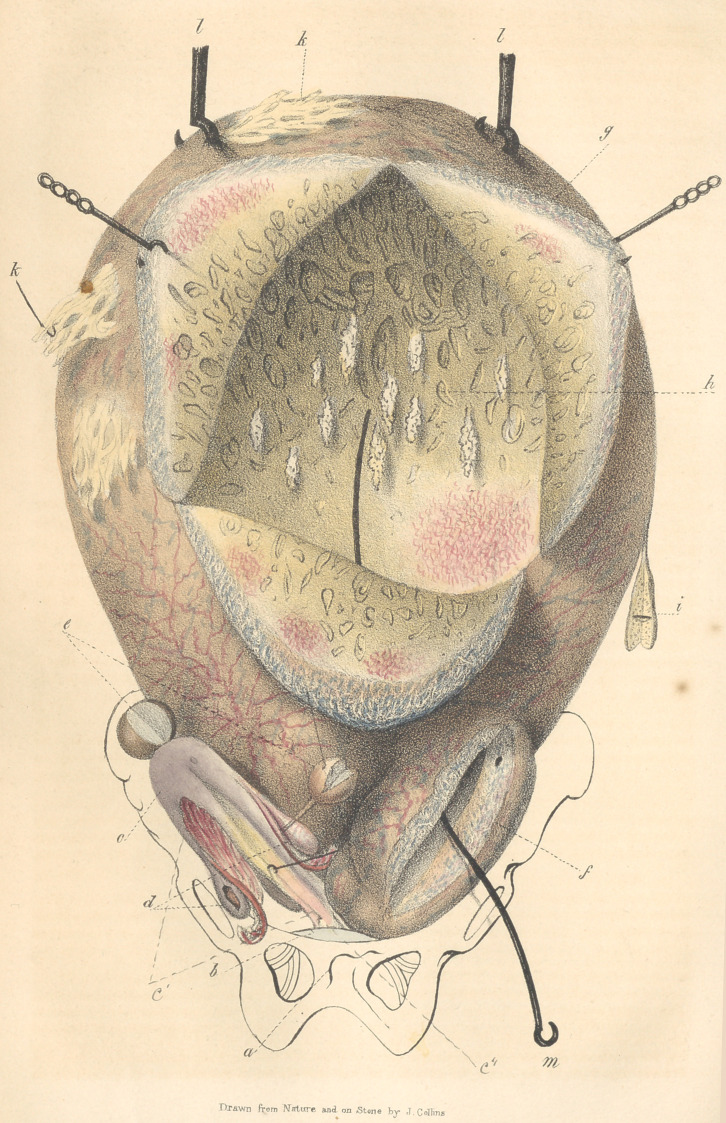# Case of Abdominal Tumour (Fibro-scirrhous) Connected with the Uterus

**Published:** 1839-07-27

**Authors:** C. W. Pennock

**Affiliations:** Physician to the Philadelphia Hospital, Blockley


### MEDICAL EXAMINER.

DEVOTED TO MEDICINE, SURGERY, AND THE COLLATERAL SCIENCES.

No. 30.] PHILADELPHIA, SATURDAY, JULY 27, 1839. [Vol. II.

*Case of Abdominal Tumour (Fibro-scirrhous) connected with the Uterus. Autopsy and Remarks by* C. W. Pennock, M. D., Physician to the Philadelphia Hospital, Blockley. (*With a Coloured Plate.*)

To the Editors of the Medical Examiner.

Gentlemen:—During the autumn of 1838, a negress entered the black women's medical ward of the Philadelphia Hospital, presenting an enormous distention of the abdomen from an internal tumour. The case excited much interest, and was regarded as one of ovarian dropsy; but since the autopsy has shown this to be incorrect, and as the precise diagnosis of similar cases must be peculiarly difficult, I take the liberty of sending you the history of this, with the accompanying plate, which has been very faithfully executed by Mr. Collins.

Yours, very truly, C. W. Pennock.

Eliza Hyson, (black,) aged thirty-six, married at nineteen, has never had children, has miscarried four times; in the three first instances between the sixth and seventh months, without any known cause; in a fourth pregnancy, fourteen years since, in the fifth month of gestation, was severely beaten and kicked in the lumbar region, which was followed by abortion the next morning. Since this event, she has not been pregnant; the menstrual function, however, has continued until the last three months; no pain was experienced at the usual menstrual period, and the appearance of the secretion was natural. Twelve years since, (two years after the beating,) a distention by a tumour in the right lumbar region was observed, which was mistaken for pregnancy; this tumour has gradually increased in size, and now presents an enormous enlargement. It has never been attended with pain, and she came into the hospital in consequence of the weight and inconvenience of the tumour, rather than for any other cause. Transient œdema of the limbs occurred in 1838. In the autumn of 1838 she entered the hospital, and was placed in the wards of Dr. Dunglison, where she remained some months. Being somewhat relieved, she requested her discharge; and, after a short absence, returned, the size of the tumour being much augmented. A few days after her re-entrance, the patient presented the following symptoms:

*February 14th.—Present state.* Slight emaciation; nothing peculiar in the expression of the countenance; intelligence perfect; no cellular infiltration; skin natural; decubitus dorsal on the left side; position in bed slightly elevated. *Chest*—well formed. Percussion preternaturally resonant, and respiration feeble beneath right clavicle—elsewhere normal. *Percussion of heart* shows it dilated; rhythm of heart nearly normal; slight bellows sound accompanies the first, heard beneath the cartilage of left third rib, and beneath the cartilages of second and third ribs on the right side, near the sternum, Pulse eighty, easily excited, somewhat tense. Abdomen enormously distended by an internal tumour; the measurement from the symphysis of pubis to the ensiform cartilage, three feet; circumference round the umbilicus four feet eight inches. Percussion is flat, with the exception of right lumbar region near the spine, where it is resonant. In the epigastric and upper part of umbilical regions, abdomen soft, elsewhere hard; hard globular masses, resisting pressure, felt in different portions of abdomen, particularly in the hypogastric, right iliac, and lumbar, extending up to right hypochondriac; fluctuation caused by palpitation on left side of tumour—none anteriorly, imperfectly felt on right side. Appetite good; constipation; some difficulty in urining; pulsation of femoral arteries distinct, but feeble.

In examination per vaginam, the finger is introduced with difficulty, from pressure of tumour filling the greater part of the cavity of the pelvis; the os tincæ, found towards the right iliac crista, soft, and unchanged—neck not obliterated. The tumour, by strong pressure, may be raised, but, upon withdrawing the hand, it sinks heavily downwards.

Treatment, palliative: mild cathartics; simple, nutritious diet; hip baths; fomentations to abdomen, &c.

On the 20th, fluctuation was observed in the upper and lateral portions of the abdomen, conveying the sensation of the existence of a slight effusion of fluid between the external parietes and tumour; no pain on pressure; pulse rather more tense, ninety per minute; skin of natural heat. Patient was directed to drink an infusion of juniper berries. ℞. Bacc. juniperi ℥j., bitart. potassæ ʒij., aqua Oj. in the day, and pulv. Doveri gr. viij. at night. The fluid diminished very sensibly in a few days. No marked fever was at any time observed; patient remained almost constantly in a recumbent posture, not, as she frequently stated, from pain, but in consequence of the weight and sense of distention when sitting. Emaciation and debility rapidly increased.

Absence from the city prevented my seeing the patient during the last week of her life. My friend Dr. Barnes, resident physician, reports, that on the 1st of March, she had a severe chill, followed by fever, pain in the abdomen, great dyspnœa, and the physical signs of peritonitis and pneumonia. All means of relief proved unavailing, and this acute attack caused death in less than thirty-six hours from its commencement.

*Autopsy fifty hours after death.*—Frame, medium size; much emaciation; no effusion into cellular tissue. Abdomen greatly enlarged, of an irregular globular form, measuring thirty-one and and a half inches from pubes to ensiform cartilage; circumference over umbilicus, where distention is greatest, fifty-one inches. Percussion of abdomen flat, except in epigastric region, where it is resonant. Abdomen soft, except in hypogastric and right lumbar regions, where a hard, irregular, semicircular mass is felt, resembling a fœtus at term; a globular mass is also felt to the left of umbilicus; fluctuation by palpitation in umbilical region.

Opening the abdomen, two quarts of fœtid, bistre coloured fluid, escaped. Peritoneum and omentum thickened, covered with numerous bright scarlet patches, and are firmly united by bands to contiguous organs. Raising the omentum, a large globular tumour is seen, by which the intestines are displaced, and forced into the epigastric region; this tumour, sixteen by fourteen inches, occupies the whole of the hypogastric, umbilical, and greater part of the lumbar regions, and is anterior to the uterus, to the body of which it is firmly connected. United to this large tumour at its inferior and left lateral margin is another tumour, which projects into the cavity of the pelvis, and rests principally in the left iliac fossa. The tumours are firmly attached to the parietes of the abdomen and pelvis by membranous bands, and are covered externally by the peritoneum, which is much thickened, of a dark red colour, and interspersed with patches of minute arterial vessels. The large tumour on its right lateral margin, is united in the extent of two inches with the cellular coat of the fundus and body of the uterus. Cellular tissue connects the peritoneal coat with the proper capsule of the tumours, which is of a pearl colour, hard, fibro-cellular, and a line in thickness. Near the connection of the tumour with the uterus, cellular tissue is very abundant; it contains numerous meshes of blood-vessels, principally veins—is deeply injected, and resembles muscular fibre. The tumour, somewhat irregular and lobulated, is of unequal firmness—in some spots soft to the touch, in others hard and resisting; evident fluctuation exists over the softer portions, corresponding with that observed during life. Incision being made into the large tumour, it is found to be filled with more than six gallons of fœtid, yellow-brown, (cafè-au-lait,) thick, and viscid fluid, in which float yellow flocculi and small fibrous masses. The walls of the tumour, from three lines to three inches thick, are of variable consistence, which, in some parts, resembles that of cartilage and grating under the scalpel—in others, the firmness of pork. General aspect of its surface, when cut, is of a light blue, passing into French gray, interspersed with pale pink, and is intersected with striæ of pearly whiteness: these bands divide it into small masses, which are smooth when first cut, but soon raise in slight elevations. The internal surface of the tumour is very irregular; in its walls are cells filled with a yellow deposit, of the consistence of cheese, and numerous pendant masses, of the fibrous character and appearance above mentioned, are attached to its parietes. Some portions of the internal surfaces are much injected, of a bright arterial hue.

The tumour in the left iliac fossa is five inches by four; shape ovoid; very firm, scalpel cutting it with difficulty; solid, with the exception of a small central canal, by which it communicates with the large tumour; around this canal, substance is softened, of a yellowish-brown colour. The tumour is fibro-scirrhous, and resembles in structure the walls of the large tumour.

The *uterus*, displaced, lying towards the right crista of the ilium, irregular in shape, three inches long, two wide, hardened, and scirrhous; its walls present a very evident muscular structure, the fibres of which interlace with the capsules of the tumours; when cut, the parietes present a marbled appearance, from pale blue and straw-coloured nodules, intersected by white striæ. The *neck* is much elongated, five inches in length, and lies between the two large tumours first described. *Os tincæ* natural, soft to the touch. *Ovaries.*—*Right ovary* slightly enlarged, contains an ounce of thick, glutinous, and hard fluid; *left ovary* normal. In the broad ligament, near its fibriated extremity, are numerous deposits in the cellular tissue of small, flat, circular, carcinomatous masses.

Near the fundus of the uterus, connected with its cellular coat, and covered by its peritoneal, are three sessile, unsoftened fibro-scirrhous tumours, from the size of a hazle-nut to that of a walnut. A fourth tumour, fibro-cartilaginous externally, and containing a thick, yellow, gelatinous fluid, similar to the largest tumour, is embedded in the cellular tissue. A pedunculated tumour, two inches in diameter, is connected with the neck of the uterus by a slender stem, four inches long.

*Stomach* contracted; mucous membrane very pale; cellular and muscular coats thickened, particularly near pylosis; pyloric orifice contracted and hardened.

The *small intestines* throughout very pale; mucous coat normal; absence of red vessels in mucous tissue, but numerous vessels containing globules of yellow substance seen in the jejenum. *Mucous coat of colon* dark gray, consistence normal; cellular tissue much thickened and opaline.

*Mesenteric glands* generally normal; meso-colic hardened and scirrhous, slightly enlarged.

*Liver*, slate colour, not congested, somewhat enlarged; consistence natural; gall bladder distended by bile, of thin consistence, and of a bright lemon hue.

*Kidneys*, left atrophied, pale; bossellated, cortical substance granulated; attached to it are several hydatids, of the size of a hazle-nut. The right kidney enlarged, displaced, resting on the bodies of the vertebræ; cortical substance buff-coloured, slightly granulated.

*Spleen* enlarged, six by four inches; soft, friable; no carcinomatous deposit.

*Bladder*—parietes thinner than natural, otherwise healthy.

*Ureters* pass on either side around the semicircumference of the large tumour.

*Thorax—lungs.* The right normal, except along the upper margin of the upper lobe, where it is emphysematous. *Left*, congested, friable, not hepatized.

*Heart.* Pericardium adherent to left pleura; upon its surface are raised opaline patches; no adhesion of pericardium to the heart. Right cavities of the heart are dilated; valves of the aorta are thickened, ossific deposit on the edges; mitral valve thickened with cartilaginous deposit; parietes of right ventricle three and a half lines; left, normal.

*Brain*, not examined.

*Remarks.*—From the colour of the serous membranes, and from that of the fluid found in the cavity of the abdomen exterior to the tumour, it is evident that chronic peritonitis and ascites must have existed for some time. The symptoms occurring in the last days of life, the vivid arterial redness in patches on the peritoneum, and the engorgement of the left lung, prove the immediate cause of death to have been an attack of acute peritonitis, with commencing pneumonia.

All the tumours were evidently of the same character; the identity of the structure of the walls and internal pendant masses of the large tumour with the formation of the others, show, that originally it must have been solid throughout. The colour, pale blue, passing into gray, the granulated appearance and hardness of the surface when cut, are indicative of scirrhus; whilst the unusual development of the fibrous deposit, and the osseous and cartilaginous changes indicate the character of the formation to be mixed, fibro-scirrhous.

Several points of interest, in addition to its great size, are connected with the history of this tumour. During the long continuance of its formation, (twelve years,) the patient does not seem to have suffered the violent pain so frequently attendant upon this class of affections: only slight œdema of the limbs resulted from the pressure of the tumour upon the abdominal arteries; the menstrual functions continued unaffected until a short time before death; and although the softening of the large tumour and the formation of purulent secretion were very great, yet no marked hectic was presented.

The singular character of these heterologous formations has engaged much of the attention of pathologists, and the cause of their production is yet but imperfectly understood. It would seem that the most satisfactory theory is, that the deposit is formed in the capillary system intermediate to the arteries and veins. In the present instance, we have seen that the carcinomatous formation was observed in the cellular tissue of the broad ligament, unconnected with any secreting glands. A question of great interest is presented respecting the circulation in these tumours: by many it is thought that the small quantity of blood which permeates them, must be from the veins. This theory, Berard, in some very happy experiments on encephaloid formation, (detailed in Dictionaire de Medecine, Art. *Cancer*,) disproves, showing it to be exclusively arterial, and that the veins become obliterated by the new formation obstructing their calibre. Anxious to ascertain whether any vessels entered into the scirrhous masses, repeated attempts at injecting them were made, in which I was very kindly and skilfully assisted by Dr. McKee, of North Carolina, resident physician. Unfortunately for a satisfactory result, the tumours had been removed sometime from the body previous to the attempt; every precaution, however, was taken to insure success, but we could not force a minute injection into the arteries further than a short distance beyond the capsule of the tumours, whilst the vessels seemed effectually closed by that tunic. The problem respecting the circulation through these formations is extremely interesting, and it is hoped that it will soon be satisfactorily elucidated.

Great pains were taken to inject the pedunculated tumour, supposing that its pedicle must contain blood-vessels for its nutrition. In this we were entirely unsuccessful; and, examining the stem by a microscope, we were convinced that it was a duplicature of the peritoneum, containing merely capillary vessels. We were induced, therefore, to regard the pedunculated tumours as resulting from a deposit in the cellular tissue beneath the peritoneum, which formation, by its growth, forms a mass, the weight of which carries before it a portion of the serous coat as one of its investing tunics, whilst the pedicle (a duplicature of peritoneum) is elongated in proportion to the increase of the tumour.

The diagnosis of the precise character of abdominal tumours connected with the uterus, is attended with much difficulty: so many of the physical characters are common to the form of tumours described in this case and to those of the ovaries, that the positive diagnosis would seem impossible. It has been supposed that in all cases of ovarian disease the menstrual function would be suppressed, and that the continuance of the catemenia would indicate that the tumour was unconnected with the ovaries; but numerous cases could be cited which entirely disprove that idea.

Among the more recent writers, the distinguished pathologist, Dr. Bright, reports several cases of ovarian tumours occurring under his own observation, where the catemenia regularly occurred at each monthly period. "No certainty," observes Dr. B., "is to be derived from this indication, as in ovarian disease the catemenia are sometimes regular, sometimes irregular, sometimes wanting; alteration in the mammæ are alike uncertain." *

The origin of these tumours from the pelvis generally distinguish them, at the commence-

* Guy's Hospital Report, 1838.

ment, from all other abdominal tumours, except those arising from the thickening of the coats of the bladder and the scirrhous affections of the uterus. In these cases, examination by the vagina must be called into requisition, and the central situation of these viscera, the pecular hardening and irregularity of the uterus, are generally sufficient to indicate the organ affected. The tumours, when softened and admitting of fluctuation, or when the cysts in the ovaries are distended by fluid, may, of course, be distinguished from ascites, by their circumscribed extent.

The ultimate prognosis of the disease we have presented, is most unfavourable. Recurring to the paper of Dr. Bright, we find twenty-one cases of malignant ovarian tumour which terminated fatally. Of these, the immediate cause of death is given in fifteen instances; in two, death took place a short time after the development of the disease, from irritation, probably induced by the mechanical pressure of the tumour; in six cases, from inflammation, caused by paracentesis; four other cases where the malignant disease undermined the constitution, and gradually led to a fatal result; three, where internal rupture of the cysts occurred.

The duration of the disease is various, from a few months to fifteen or twenty years. As regards the *cure*, no flattering prospect can be presented. In the early stage of the disease, before the formation has attained a large size, occasional local depletion, by cups and leeches, the *careful* exhibition of iodine, with its combinations, together with its application by inunction—revulsives, rigid observations of all hygienic rules, "so as to maintain the general health in a state unfavourable to the rapid development of the disease," are all that experience has taught us to expect from our remedies. The indication is, therefore, to restore and preserve the natural secretions, maintain the strength, and subdue inordinate action, whether local or general.

It is of the first importance that the patient should have the benefit of a pure atmosphere—crowded cities, and more especially the wards of hospitals, are objectionable. Of the therapeutic remedies calculated to effect the indication, and to relieve pain, irritation, and the harassing neuralgic and dyspeptic symptoms which are the general attendants upon the disease, may be mentioned, local application to the spine, either as counter-irritants or anodynes, and the exhibition, among others, of the bitter and mild tonics, alkalies, antacids, taraxicum, minute doses of the mercurials, sarsaparilla—the various narcotics, as conium, hyosciamus, stramonium, belladonna, opium, with the salts of morphia, whilst care must be taken to prevent constipation, by the administration of the mild laxatives.

By Dr. S. Young, of London,* pressure in cases of external scirrhus has been very highly recommended, and his views of its beneficial effects have been entirely confirmed by Recamier, one of the physicians of the Hotel Dieu of Paris. It is impossible to produce much compression of internal abdominal scirrhous tumours, yet, relying on the authority of the distinguished names mentioned, it is proper that tight bandaging of the abdomen should be employed.

Of the internal remedies, Recamier* places his great reliance upon conium, the curative virtues of which greatly depend, he states, on the quantity of food consumed by the patient; that is to say, the operation of the remedy was much more marked when but a small quantity of food was allowed, whilst its effects were hardly perceptible when the quantity was considerable. Whilst using the conium, he therefore restricts his patients to a severe diet. The following are the principles of his treatment:†

1st. The patient takes a dose of the extract of conium,‡ evening and morning, two hours before the first, and two hours before the last meal. The amount of the first dose is half a grain, which is gradually increased to six grains each time. This dose is continued for a fortnight, in order that the organs may become habituated to its operation, and is afterwards increased to twelve grains each time, beyond which it is not necessary to carry the remedy, as its influence is then sufficient. The twelve-grain dose is continued from two to three or four weeks.

2d. After each dose of the conium, as well as at meals, the patient uses a decoction of sarsaparilla, (composed of two ounces of the root to two pounds of water,) instead of water.

3d. Only the third of the ordinary quantity of food is allowed, which ought to be very simple, and divided into three small meals.

4th. If the conium disagree in one form, it should be given in another, or the aconitum may be used instead, but in lesser quantity than the conium. Towards the end of the treatment, the dose of the conium is gradually diminished and the diet gradually increased.

By these remedies, judiciously employed, M. Recamier states his success in cancer to have been very satisfactory. In this he has been more fortunate than most physicians have been in their treatment of the malignant abdominal tumours connected with the uterus: generally, all they effect is, to retard the progress of the disease, render it stationary for a time, but sooner or later it recurs with renewed violence and goes rapidly forward.

When the disease has advanced to softening, and the fluctuation is distinctly felt, paracentesis

*Cases of Cancer, &c., London, 1816.

* Recherches sur le traitment de Cancer, par M. Recamier.

† Cyclopedia of Practical Medicine, Art. Scirrhus.

‡ M. Recamier prepares the extract of conium in the following manner, and to the excellence of the preparation he ascribes much of his success. "The plant is submitted to the action of the vapour of vinegar or alcohol, before the juice is expressed from it; the juice is afterwards exposed to the heat of a sand-bath, and evaporated to the consistence of an extract. The extract thus obtained has not the nauseous odour of that usually employed, whilst it possesses all the deobstruent virtues, and sits better on the stomach than the latter."

Drawn from Nature and on Stone by J. Collins is recommended, which operation is performed in the hope of prolonging life—cure in this case cannot be anticipated.

*Explanation of the Plate.*

The tumour, one third of the natural size, is represented as raised upwards from the pelvis, by the hooks *l, l.*

*a.* Pelvis.

*b.* Urinary bladder.

*c.* Body of the uterus.

*c'.* Neck of the uterus, which is much elongated, (5 inches.)

*c''.* Os tincæ.

*d.* Ovaries and fallopian tubes.

*e.* Tumours, sessile and pedunculated.

*f.* Fibro-scirrhous tumour, connected with the large tumour, g, with which it communicates, as indicated by the wire *m.*

*g.* Large tumour laid open by a triangular incision.

*h.* Internal surface, very irregular from the pendant scirrhous masses and numerous cells in the walls of the tumour—on the smoother portions are patches of arterial capillaries.

*i.* Fibrous pedunculated tumour, composed principally of ossific deposit.

*k, k.* Membranous bands passing to omentum and peritoneum of contiguous organs.

## Figures and Tables

**Figure f1:**