# Errata

**Published:** 1839-08-03

**Authors:** 


Errata.—In pages 469 and 470, for "palpitation," read *palpation*.

In second column of page 470, 29th line, instead of "hard fluid," read *viscid fluid*.

In page 471, 20th line of second column, for "vessels," read *veins*.

In our last number, the quantity of serum found in the ventricles of a hydrocephalus was stated to be three ounces; it should have been fifty-two ounces. Mistakes of this kind are so rarely made by our printer, that we can readily point them out.

